# Instar determination, development, and sexual dimorphism for *Gynaephora menyuanensis* (Lepidoptera: Lymantriinae) and ultrastructure of adult antennae

**DOI:** 10.1093/jisesa/ieaf006

**Published:** 2025-03-14

**Authors:** Hainan Shao, Chen Yuan, Yunxiang Liu, Xin Xin

**Affiliations:** State Key Laboratory of Plateau Ecology and Agriculture, Academy of Agricultural and Forestry Sciences, Qinghai University, Xining, Qinghai, China; State Key Laboratory of Plateau Ecology and Agriculture, Academy of Agricultural and Forestry Sciences, Qinghai University, Xining, Qinghai, China; Provincial Key Laboratory of Agricultural Integrated Pest Management in Qinghai, Academy of Agricultural and Forestry Sciences, Qinghai University, Xining, Qinghai, China; State Key Laboratory of Plateau Ecology and Agriculture, Academy of Agricultural and Forestry Sciences, Qinghai University, Xining, Qinghai, China; Provincial Key Laboratory of Agricultural Integrated Pest Management in Qinghai, Academy of Agricultural and Forestry Sciences, Qinghai University, Xining, Qinghai, China; Department of Crop Soil Sciences, Washington State University, Pullman, WA, USA

**Keywords:** life history, morphology, antennal sensilla, grassland caterpillar, Qinghai-Tibet Plateau

## Abstract

*Gynaephora menyuanensis* Yan & Zhou is one of the most devastating pests that harm the ecosystem of alpine meadows and hinder the advancement of animal husbandry. However, the current knowledge of the morphology of the different developmental stages within *G. menyuanensis* reveals an information deficit that needs to be addressed. This study is the first to report the life history, sexual dimorphism, and morphology of eggs, mature larvae, pupae, and adult antennal sensilla types of *G. menyuanensis*. This study used a *K*-means clustering method, based on the head width, body length, body width, and the number of crochets of larvae at each instar, to differentiate instars of *G. menyuanensis*; the description of the morphology of larvae, pupae, and adult antennae employed light microscopy and scanning electron microscopy photographs. The results revealed that the instar grouping was reliable and verified by the Brooks-Dyar combined with Crosby rules, revealing that the larval stage of *G. menyuanensis* comprises 7 instars. This species produces one generation per year in the alpine meadow, with its life cycle lasting approximately 300 d in total. The pupae and adult antennae significantly differed between the sexes, indicating sexual dimorphism in the 2 genders. Nine types and 14 subtypes of antennal sensilla were observed in male antennae (bipectinate), while only 3 types and 3 subtypes were found in female adult antennae (club-like). Our findings have implications for better understanding the life history, adaptation strategies under extreme environmental conditions on the Qinghai-Tibet Plateau and developing scientific and effective pest control methods.

## Introduction

*Gynaephora* (Lepidoptera: Erebidae: Lymantridae) is a small genus primarily occurring in the high mountainous areas of the Northern Hemisphere and the Arctic tundra (Zhou and Yin 1979, Yuan et al. 2015, Zhang et al. 2017a, Lv et al. 2023). At present, 15 species of *Gynaephora* have been identified worldwide, including 8 in China which are endemic to the Qinghai-Tibet Plateau (QTP) (Zhou and Yin 1979, Zhang and Yuan 2013, Yuan et al. 2015). *Gynaephora menyuanensis*Yan & Chou (1997) originated from Menyuan County, Qinghai Province. It is distributed in the northeast of Qinghai Province and parts of Gansu Province (Zhou and Yin 1979, Zhang et al. 2017a). It has caused widespread concern as the most important pest in alpine meadows, which can survive in the extreme environment of the Tibetan Plateau (Zhang et al. 2017a). The caterpillar stage is characterized by numerous bristles densely covering the whole body, causing cutaneous pruritus in people and oral mucosal ulcers in yarks. The larvae mainly feed on more than 20 forage grasses, including *Elymus nutans* Griseb.*, Stipa capillata* L*., Artemisia lancea* Vaniot*, Poa araratica* Trautv*.,* and *Krascheninnikovia ceratoides* (L.) Gueldenst. (Zhang et al. 2017a), and change the plant community structure of grassland, thereby aggravating the deterioration of the grassland ecological environment and causing livestock poisoning, which seriously impedes the healthy development of animal husbandry on the QTP (Wang et al. 2023a). For effective pest control, applications must be timed to coincide with the period when instars are the most vulnerable (Cen et al. 2015). Determining larval stage and distribution is important for life table analysis, key factor analysis, pest control, and entomological studies (Peterson et al. 2019, Wang et al. 2022).

In general, the determination of the larval instar stages depends on morphometry protocols (such as head width) because holometabolous insects rarely exhibit instar-specific traits (Bharti et al. 2023, Lambiase et al. 2024, Mohanty et al. 2024). Distinctive size groups may be determined by the statistical grouping of larval measurements. The presumed instar number is subsequently validated by applying the Brooks-Dyar Rule which states that any highly sclerotized structure increases in size in each subsequent instar by a constant growth ratio for the average sizes of each group (Floater 1996, Peterson et al. 2019). However, the overlapping size-frequency distributions make instar numbering difficult (Hunt and Chapman 2001, Wu et al. 2013). Multiple morphometric characteristics are involved across studies, resulting in inconsistency across size distributions based on one character and those made based on another (Wang et al. 2022). Consequently, cluster analyses have been proposed to address such problems, including linear discriminant analysis (LDA) and *k*-means clustering. *K*-means clustering, the most commonly used algorithm for this purpose, has been employed to determine the instar stages of insects (Yang et al. 2018, Zheng et al. 2019).

Given the obvious sexual dimorphism in *G. menyuanensis*, studying the olfactory sensory system of the 2 sexes is key in mate searching and reproduction (Zhou and Yin 1979). The antennae are the primary sensory organs, essential for mechanoreception, gustation, olfaction, temperature, and humidity perception in Lepidoptera (Abd El-Ghany and Faucheux 2021, 2022, Rani et al. 2021). The sensillum is the basic structural and functional unit of insect sensory systems. Morphologically, sensilla can be classified into various types: trichoid, basiconic, chaotic, coeloconic, and styloconic sensilla; functionally, they may represent mechano-, chemo-, hygro-, and thermoreceptors (Wang et al. 2023b, Yamany and Abdel-Gaber 2023, Abd El-Ghany et al. 2024). Sexual dimorphism (eg morphological difference, variations in sensilla shape, numbers, and distribution of each type of sensilla) has been reported in other members of Erebidae. For instance, Silava et al. (2019) studied the antennae of male and female *Automeris liberia* Cramer and reported sexual dimorphism in the antennae of males (bipetectinate) and females (filiform), and the sensilla trichodea were more abundant on male antennae than on those of females. Zheng et al. (2023) report that the female antennae of adult *Paranthrene tabaniformis* are clavate, while male antennae are pectinate, and sex-specific differences were found in the ultrastructure of each type of sensilla. In recent years, sex pheromones—based pest management technology plays a key role in monitoring and controlling insect pests in the agricultural, forestry, and public health sectors (Wang et al. 2023a, Yang et al. 2024). Therefore, studies on the structures of sensilla in insects are important to help elucidate the olfactory mechanisms and in the recognition of electrophysiologically active molecules.

*Gynaephora menyuanensis* is a polyphagous pest with particular life history characteristics adapted to its habitat and has developed ecological countermeasures (Yan 2006). The purpose of this study is to clarify the larval instar division, life history, and adult ultrastructure of the sensilla on the antennae of male and female *G. menyuanensis*, aiming to elucidate the survival strategy of this pest throughout its life cycle and the contribution of life history characteristics at each stage to population growth and maintenance. These results can help clarify the underlying mechanisms of sexual selection involved in *G. menyuanensis* and provide a theoretical basis for further study of population dynamics, population regulation mechanisms, and effective control of this pest.

## Materials and Methods

### Insect Collection and Rearing

Larvae of different instars were collected from Menyuan County, Qinghai Province, China from March to October during 2020 to 2023. The specimens were brought back to the laboratory and were reared on fresh grass (*Stipa capillata* L. and *Artemisia lancea* Vaniot) in plastic boxes (8 cm diam., 20 cm ht.). All collected samples were kept in an incubator at 15.0 °C ± 3.8 °C with a relative humidity of 65% ± 10%. The molt and pupation times were recorded daily. Ten healthy female and male adults were randomly selected for further testing after eclosion.

### Measuring Larval Instar

For morphometric measurements, healthy larvae were randomly selected and quickly euthanized using 75% alcohol. A Nikon SMZ1500 stereomicroscope (Nikon Corporation, Tokyo, Japan) was used to measure the head width, body length, body width, and number of crochets of the larvae (Supplementary Fig. S1). Data were analyzed using the R software (version 3.3.2). *k*-means clustering was provided by the “stats” package, and Hartigan and Wong’s algorithm was utilized as the default setting. Subsequently, exponential-progression equation fitting was assessed using the “nls2” package. “ggplot2” and “Rmisc” packages were used for graphical analyses. The Brooks-Dyar and Crosby rules were employed to estimate the datasets calculated through *k*-means clustering. The relationship between the measurements of morphological characteristics *y* with the instar number *×* is given by


$$\begin{array}{l} y=ae^{b}x \end{array}$$

where$e^{b}$is the growth-ratio constant, and *a* is the theoretical size corresponding to 0 instars. The Crosby ratio represents the difference between consecutive growth ratios (Yang et al. 2018).

The Brooks index, Crosby index, and linear regression (Zheng et al. 2019) were applied to verify the rationality of instar division (Loerch and Cameron 1983). When the absolute value of the Crosby index is less than 0.1, the age division index is considered reasonable, and vice versa. The Brooks index is calculated as follows:


$$\begin{array}{l} bn=X_{n}/X_{n-1} \end{array}$$

where *X*_*n*_ and *X*_*n-1*_ represent the mean values of each measured value of *n*-instar and *n-1* instar larvae, respectively; *n* represents larval instar.

The Crosby index is given by


$$\begin{array}{l} C_{n}=\;\left(B_{n}-B_{n-1}\right)/B_{n} \end{array}$$

where *B*_*n*_ and *B*_*n-1*_ represent the Brooks index of *n* instar and *n-1* instar, respectively; *C*_*n*_ represents the Crosby exponent.

### Life Cycle of G. *menyuanensis*

During 2020 to 2023, egg masses of *G. menyuanensis* were collected from habitats in Menyuan, Qinghai Province, China. The egg masses were then brought to our laboratory. After dipping in 5% formaldehyde solution for 30 s, the eggs were washed with distilled water and dried at 20 ± 1 °C. Ten eggs were placed in a sterilized petri dish (Ø 9.0 cm) with a humid filter paper to maintain freshness. Specifically, 100 Petri dishes with a total of 1000 eggs were incubated in a growth chamber (20 ± 1 °C) and observed every 12 h. When the eggs hatched, the first instar larvae entered diapause. To overwinter, larvae in the same dishes were placed in insect-rearing cages under grass outdoors until the following March. Then, the cages were returned and reared in dishes with *Festuca rubra* and *Poa pratensis* L. in a growth chamber (20 ± 1 °C). The hatching rates, larval survival, body size, and time of larval molts and pupation were recorded.

### Preparation of the Antennae for Scanning Electron Microscopy

The antennae of 10 female and 10 male adults of *G. menyuanensis* were carefully excised with a scalpel under a stereomicroscope and then fixed in 2.5% glutaraldehyde for at least 12 h, followed by washing thrice with 0.1 mol·L^-1^ (pH 7.2) phosphate buffer (Zacharuk 1980). After dehydration in an ethanol gradient for 20 min at each concentration (30%, 50%, 70%, 80%, 90%, 95%, and 100%), the samples were freeze-dried for 4 h, sputter-coated with gold, and then assessed under a scanning electron microscope (JEOL 7900F) at 5 kV (Jiang and Hua 2015). ImageJ 1.49 was used to measure antennal length, sensilla length, and base diameter.

### Statistical Analyses

Values of morphological characteristics for different larval instars, as well as the length and width of the antennae and all sensilla of female and male adults, are presented as means ± SE. The head width, body width, body length, and number of crochets of different larval instars were compared using a 1-way analysis of variance (ANOVA) and LSD test (*P* < 0.05). The data were checked whether the homogeneity of variances was satisfied before conducting ANOVA using Brown-Forsythe test. Then, we filtered some outliers to make sure all the data followed the normal distribution and had homogeneity of variances. The box plot was created by analyzing each instar per variable, where dots represent different feature values. The number of instars can be confirmed when the feature values for each instar show a clustered distribution with no overlap between contiguous instars. A linear discriminant analysis was performed to evaluate how these 4 measurements varied among larval instars (Chen and Seybold 2013). The means of each instar per variable were plotted against instar number. A fitted curve suggested that a consistent geometric progression occurred during development and no instar was overlooked. We employed a Student’s *t*-test to compare the lengths and widths of the antennal segments between female and male adults. The experimental data were analyzed by SPSS 27.0 package and Origin 9.0. Sensilla were classified based on the external morphology and surface features according to Schneider (1964), Zacharuk (1980) and Faucheux (1990).

## Results

### Larval Instar Division of *G. menyuanensis*

**Fig. 1.**
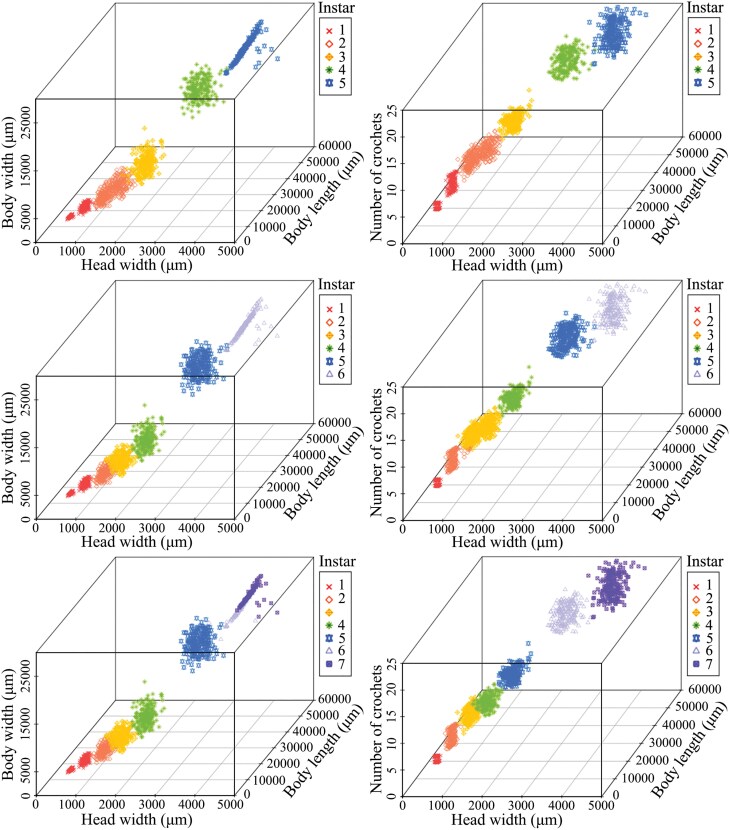
Instar determination of *Gynaephora menyuanensis* larvae by *k*-means clustering, where A, B, and C are the 5-, 6-, and 7-centroid runs, respectively. Different colors represent different groups.

**Fig. 2.**
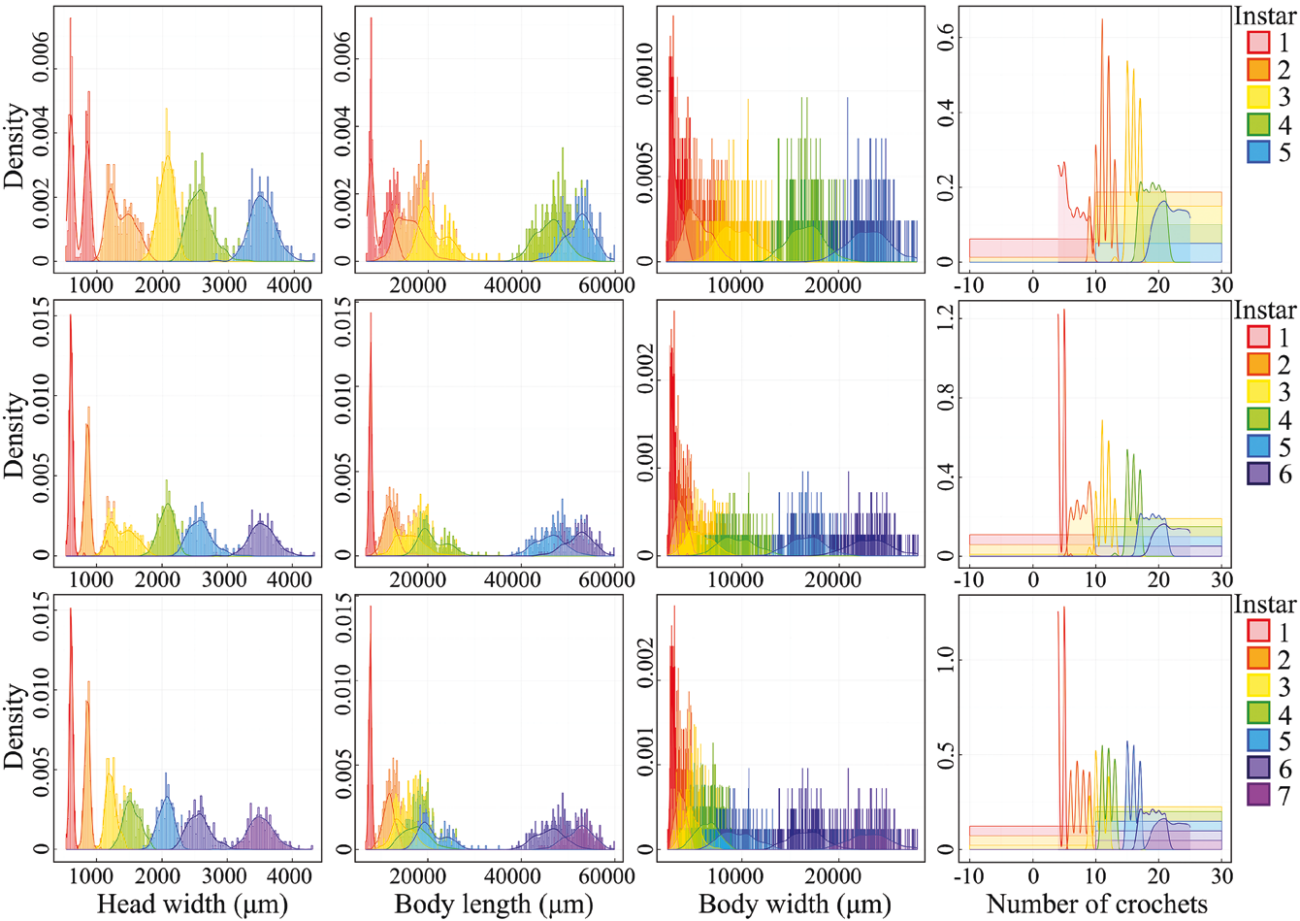
Size-density distribution histograms of instar groupings based on head width, body length, body width, and number of crochets. The density curves of each group are fitted and annotated by colors. The size groups are arranged by rows ascending from top to bottom.

In this study, 1500 larval *G. menyuanensis* were used to confirm their morphological identity. The head width, body length, body width, and number of crochets were measured. According to the multivariable scatter plots and density-distribution histograms resulting from *k*-means clustering, 2 of the run centroids, 6 and 7, were recognized as the least overlapping, separate clusters (Figs. 1 and 2). The parameters i.e., *a, e*^*b*^, goodness-of-fit [*r*^*2*^], Crosby ratios, and the residual sum-of-squares (RSS) were calculated using the Brooks-Dyar equation, plotted by the group averages of each clustering event (Table 1). The RSS and goodness-of-fit values demonstrated that the average size under the 7-instar hypothesis correlated strongly with the Brooks-Dyar rule. The combined parameters revealed that the growth trajectories of the 7-instar hypothesis aligned with the Brooks-Dyar rule. As shown in Table 2, significant differences were observed among different instars for the same index (*P *< 0.05). The Brooks index (*bn*) and Crosby index (*Cn*) were employed to test the rationality of instar division. The *Cn* for larva head width was less than 0.1 (Table 2), and the *Cn* for body length, body width, and number of crochets exceeded 0.1. Therefore, head width combined with body length, body width, and number of crochets can be used as vital indexes for age identification.

**Table 1.** Five to 7 instar-grouping results (including the variables head width, body length, body width and crochets number) for *Gynaephora menyuanensis*, as analyzed by the Brooks-Dyar and Crosby rules.

Variable	Parameter	Five instar	Six instar	Seven instar
Head width	A	548.556	453.509	483.183
	*e* ^*b*^	1.482	1.423	1.330
	*r* ^*2*^	0.913	0.963	0.984
	Crosby ratio	6.420	7.766	7.289
	RSS	42.005	17.966	7.847
Body width	A	1,963.106	1,600.093	1,750.229
	*e* ^*b*^	1.664	1.559	1.429
	*r* ^*2*^	0.936	0.935	0.942
	Crosby ratio	11.769	14.439	13.201
	RSS	50.135	51.167	45.453
Body length	A	615.500	504.863	553.796
	*e* ^*b*^	1.567	1.489	1.373
	*r* ^*2*^	0.913	0.935	0.925
	Crosby ratio	8.500	10.363	9.447
	RSS	55.243	41.032	47.770
Crochets number	A	4.844	3.985	4.228
	*e* ^*b*^	1.401	1.366	1.286
	*r* ^*2*^	0.809	0.907	0.921
	Crosby ratio	4.540	5.519	5.201
	RSS	76.383	37.131	31.597

**Table 2.** Measurements of morphological characteristics in different instars of *Gynaephora menyuanensis.*

Measured parts	Instar	Measurements	Range	*F*	df	*P*	Index *b*n	Index *C*n
Head width (mm)	1	0.608 ± 0.002 g	0.538–0.692				—	—
	2	0.855 ± 0.003 f	0.726–0.979	124.09	6, 1050	< 0.001	1.406	—
	3	1.210 ± 0.005 e	0.997–1.404				1.416	0.007
	4	1.596 ± 0.009 d	1.427–1.887				1.319	-0.074
	5	2.062 ± 0.008 c	1.911–2.278				1.342	0.017
	6	2.575 ± 0.013 b	2.306–3.106				1.249	-0.074
	7	3.522 ± 0.014 a	3.122–4.095				1.368	0.087
Body length (mm)	1	2.849 ± 0.015 g	2.315–3.394				—	—
	2	3.692 ± 0.036 f	2.692–4.778	160.32	6, 1050	< 0.001	1.296	—
	3	4.631 ± 0.053 e	2.735–6.573				1.254	-0.034
	4	6.428 ± 0.074 d	4.276–8.866				1.388	0.097
	5	9.562 ± 0.122 c	5.966–15.601				1.488	0.067
	6	16.640 ± 0.122 b	11.692–21.391				1.740	0.145
	7	23.103 ± 0.144 a	17.412–28.061				1.388	-0.254
Body width (mm)	1	0.771 ± 0.002 g	0.686–0.865				—	—
	2	1.164 ± 0.009 f	0.736–1.440	298.55	6, 1050	< 0.001	1.510	—
	3	1.470 ± 0.016 e	0.860–2.124				1.263	-0.196
	4	1.730 ± 0.019 d	1.224–2.434				1.177	-0.073
	5	2.117 ± 0.022 c	1.556–3.426				1.224	0.038
	6	4.608 ± 0.022 b	3.804–5.433				2.177	0.438
	7	5.232 ± 0.021 a	4.389–5.985				1.135	-0.918
Crochets numbers	1	4.3 ± 0.4 g	4–5				—	—
	2	7.2 ± 1.0 f	6–9	68.39	6, 1050	< 0.001	1.691	—
	3	10.3 ± 1.4 e	9–12				1.437	-0.177
	4	12.7 ± 2.1 d	11–13				1.227	-0.171
	5	16.3 ± 1.3 c	15–17				1.290	0.049
	6	19.8 ± 2.0 b	17–21				1.214	-0.065
	7	22.2 ± 3.1 a	19–25				1.117	-0.087

Note: Data within the same column and in the same measured part followed by the different lowercase letters indicated significant difference at *P *< 0.05 (One-way ANOVA).

The head width, body length, body width, and number of crochets in larvae of different instars were analyzed by regression (Supplementary Figs. S2 and S3), and the correlation coefficients (*R*^2^) of the fitted exponential regression equations were 0.9992, 0.9782, 0.9399, and 0.9964, respectively. All 4 measures showed a positive correlation with age (Table 2; Supplementary Fig. S3).

### Life History Traits of G. *menyuanensis*

**Fig. 3.**
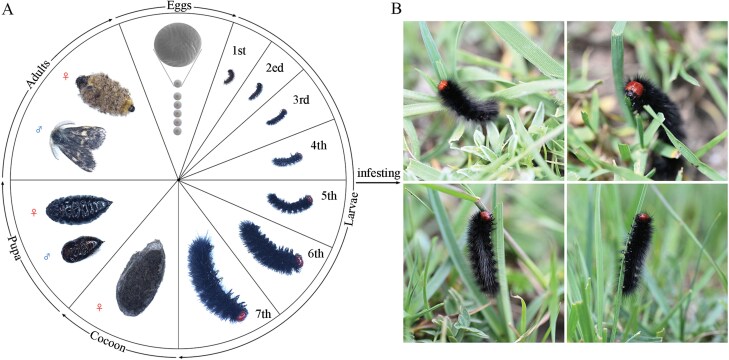
The life cycle of *Gynaephora menyuanensis.*

*Gynaephora menyuanensis* is a holometabolic insect, with one generation per year in alpine meadows. It undergoes 4 developmental stages: egg, larva, pupa, and adult (Fig. 3A). *G. menyuanensis* feeds mainly on *Elymus nutans* in the alpine meadows (Fig. 3B). When the eggs hatch, they become newly hatched black caterpillars with red heads and overwinter under the roots or in the soil for about 230 ± 5.2 d (Table 3; Fig. 3A). Larvae are gregarious in the 7 instar stages, feeding on tender leaves day and night. After shedding its shell 6 times, the black caterpillars become mature caterpillars, stop feeding, and prepare for cocooning. All development stages of *G. menyuanensis* (egg-larva-pupa) last for about 350 d (Table 3; Fig. 3A). After about 5–15 d, the pupae transform into moths and mate, with the female laying eggs in her own cocoon, dying soon afterward (Table 3; Fig. 3A). The number of eggs laid by a single female *G. menyuanensis* was approximately 100 to 120, and the eggs hatched after 20 ± 3.6 d (Table 3; Fig. 3A).

**Table 3.** Phenology of *Gynaephora menyuanensis.*

Stages	Jan.–Apr.			May.			Jun.			Jul.			Sep.			Oct.			Nov.–Dec.			Time (d)
	F	S	L	F	S	L	F	S	L	F	S	L	F	S	L	F	S	L	F	S	L	
1^st^ instar	⟐	⟐	⟐															⟐	⟐	⟐	⟐	230 ± 5.2
2^nd^ instar			⟐	⟐	⟐	⟐																14 ± 2.4
3^rd^ instar					⟐	⟐	⟐															14 ± 3.1
4^th^ instar							⟐	⟐	⟐													16 ± 2.5
5^th^ instar								⟐	⟐	⟐												15 ± 2.0
6^th^ instar										⟐	⟐	⟐										15 ± 2.5
7^th^ instar											⟐	⟐	⟐									13 ± 4.4
Male Pupae											✦	✦	✦	✦								13 ± 2.1
Female pupae												✦	✦	✦	✦							5 ± 0.7
Male adults												♂	♂	♂	♂							13 ± 2.6
Female adults													♀	♀	♀	♀						5 ± 2.4
Eggs														⚪	⚪	⚪	⚪	⚪				20 ± 3.6

Larvae: ⟐; Pupae: ✦; Adults: ♂, ♀; Eggs: ⚪;.

F: The first 10 d of a month; S: The second 10 d of a month; L: The last 10 d of a month.

Morphology of the developmental stages

Egg (Fig. 4)

**Fig. 4.**
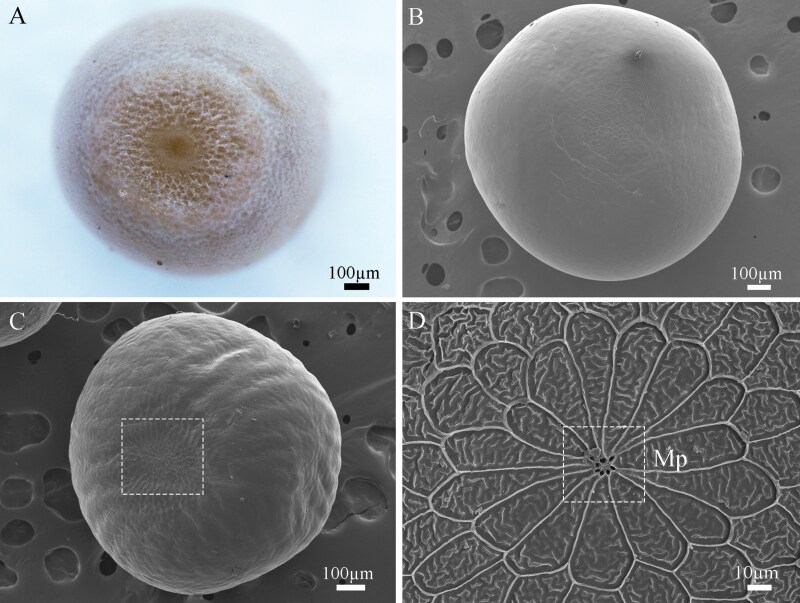
Egg morphology of *Gynaephora menyuanensis*. (A) general view; (B) upper—lateral view; (C) detail of the micropilar region, with annulus and rosette; (D) detail of the rosette. Mp: micropyle.

Newly hatched eggs are yellowish, elliptical, with a mean diameter on the longitudinal axis of 1.24 mm (*n *= 10 hatched eggs) and 1.66 mm (*n *= 10 intact eggs). The surface of the ova is covered with dense trichome (Fig. 4A). The exochorion is predominantly smooth, with a regular crown of reticulation (Fig. 4B). The micropylar is located in the center of the polar zone of ovum, with a rosette formed of 15 or 16 petal-shaped cells (*n *= 10), and 7 or 8 micropylar openings (*n *= 10). Mosaic-like projections are present on each petal of the rosette (Fig. 4C, D). The rosette is surrounded by rows of cells enclosed by faint ribs, with an approximately hexagonal shape, though rarely with a different form.

Pupa (Fig. 5)

**Fig. 5.**
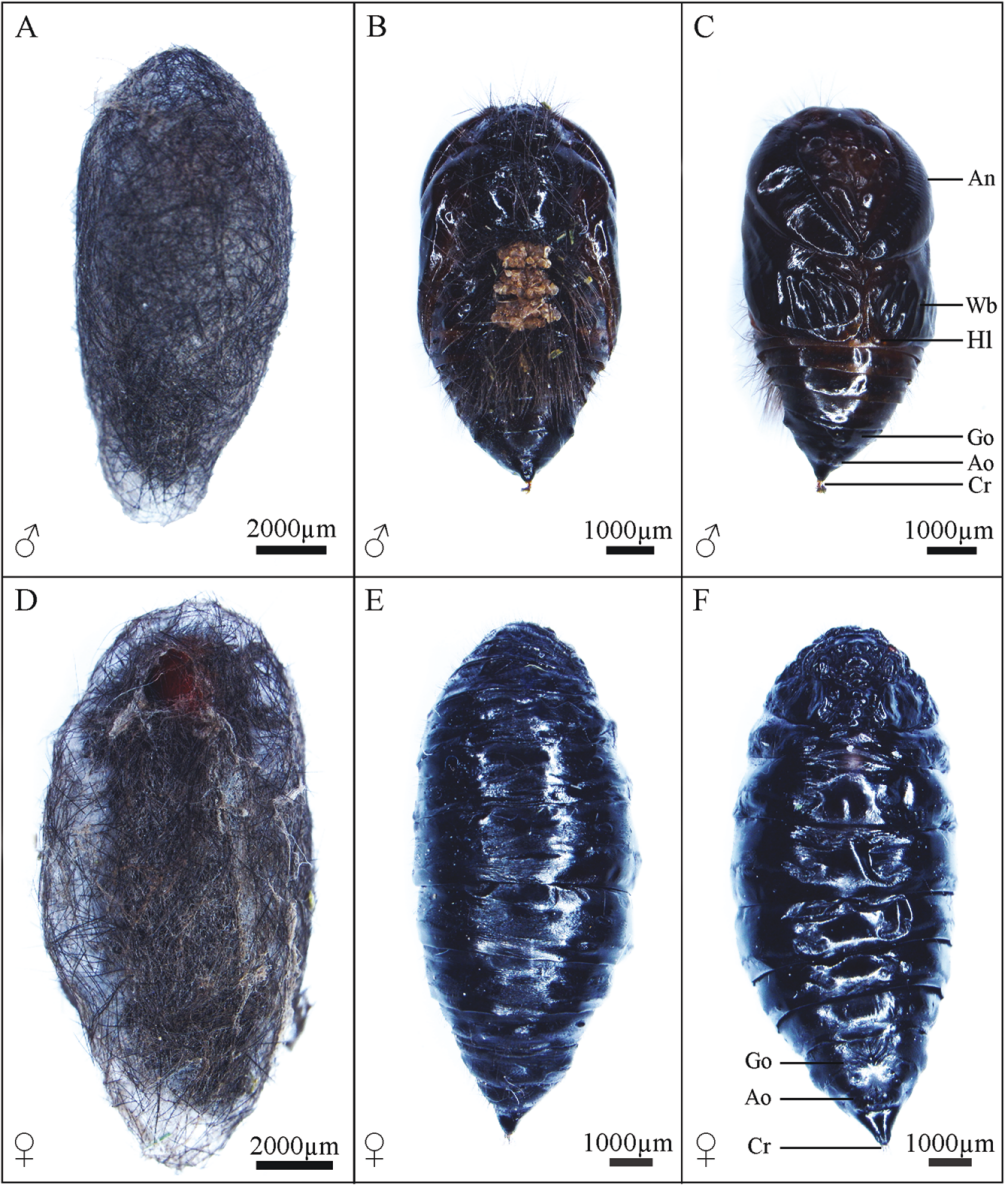
Pupae of *Gynaephora menyuanensis*. (A) **♂**, whole view; (B) **♂**, dorsal view; (C) ♂, ventral view; (D) ♀, whole view; (E) ♀, dorsal l view; (F) ♀, ventral view. An, antenna; Wb, wing bud; Hl, hindleg; Go, genital pore; Ao, anal opening; Cr, cremaster.

Pupae of both genders are covered by a black cocoon (Fig. 5A, D) with evident longitudinal retraction of the body segments. The male pupae of *G. menyuanensis* are oval and brown, with 3 yellowish crystalline glands on the abdomen, bearing numerous setae (Fig. 5B). The maxillae and legs are visible between the antennae in males, with the former being long and wedge-shaped. The abdomen has 9 segments, with segments I-VII showing 6 pairs of dioneiform organs (Fig. 5C). The male genital pore is located on the midventral area of A9 and the distal abdomen possesses 6 to 9 cremastral hooks (Fig. 5C). Their average body length is 8.91 ± 0.23 mm, and width is 4.55 ± 0.13 mm. The female pupae are fusiform, dark black, with a smooth body surface, evident eyes, mandibles and indistinctive antennae (Fig. 5E, F). The female genital pores are located along the ventral midline of A8 (ostium bursae) and A9 (ostium oviductus) and the distal abdomen bears 3 or 4 cremastral hooks (Fig. 5F). Their average body length is 13.03 ± 0.33 mm, and width is 7.56 ± 0.25 mm. Because the male pupae must differentiate wing buds and accessory organs, their pupal period is longer (about 15 d).

### Sexual Dimorphism in Adult G. *menyuanensis*

**Fig. 6.**
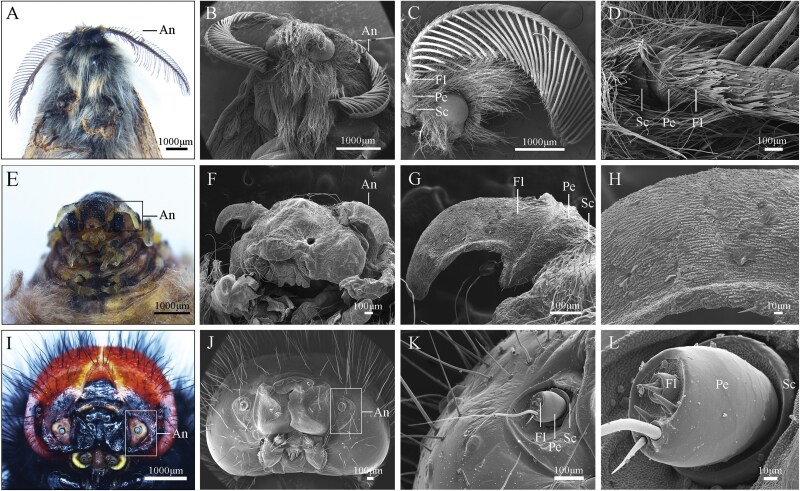
SEM image of the mature larval and adult antennae of *Gynaephora menyuanensis*. (A) Frontal view of the head of the male adult with its antennae; (B) Overall view of the male adult antennae; (C) Ventral view of the flagellum (Fl) in male adults showing a pinnate branch (Pb); (D) Basal part of the male antenna showing the scape (Sc), pedicel (Pe), and parts of flagellomeres; (E) Frontal view of the head of the adult female antennae; (F) Overall view of the female adult antennae; (G) Basal part of the female antenna showing the scape (Sc), pedicel (Pe), and flagellum (Fl); (H) A magnified view of flagellum; (I) Overall view of the head of mature larvae; (J) Frontal view. An, antenna; (K) Basal part of the larval antenna showing the scape (Sc), pedicel (Pe); (L) Magnified view of the larval antennae.

As a holometabolous insect, *G. menyuanensis* rarely exhibits instar-specific traits (Fig. 3). However, evident sexual dimorphism was established in *G. menyuanensis* adults (Figs. 3A and 6). Sexual dimorphism in overall body length and appearance is pronounced between the 2 sexes, with an average length of 11.75 ± 0.28 mm in males, and 15.60 ± 0.37 mm in females (*t *= 2.21; df = 18; *P* = 0.008). The male has 2 pairs of wings covered by slender scales, whereas the female is apterous and completely covered with golden setae. The antennae of males and females show conspicuous morphological dimorphism (Fig. 6). The antennae of male adults are bipectinate, and those of the females are club-like. The average length of male adult antennae (*n* = 10) is 6240.4 ± 250.20 μm, significantly longer than that of the female with a mean length of 1031.4 ± 18.23 μm (*t *= 2.36; df = 18; *P <* 0.05) (Table 4). The types, number, and distribution of sensilla on the antennae of the 2 sexes differ. Male adult antennae have 9 types and 14 subtypes of sensilla, while 3 types of sensilla are observed in the female adult antennae (Table 5).

**Table 4.** Mean length of each antennomere on the antennae of adult *Gynaephora menyuanensis.*

Sex	Length/μm	Scape length/μm	Pedicel length/μm	Flagellum length/μm
Male	6240.4 ± 250.20 a	152.6 ± 5.25 a	130.8 ± 3.60 a	5957.1 ± 180.66 a
Female	1031.4 ± 18.23 b	33.4 ± 2.56 b	109.1 ± 8.14 b	888.9 ± 15.53 b

Data in the table are mean ± SE.

Different letters indicate *P *< 0.05 in the independent sample *t*-test.

**Table 5.** Lengths, basal widths and morphological characteristics of sensilla on the antennae of adult *Gynaephora menyuanensis*.

Sensilla types	Gender	Length (μm)	Base diameter (μm)	Wall	Tip	Socket
BB 1	♂	116.4 ± 15.68	3.2 ± 0.18	Smooth	Sharp	Dent
BB 2	♀	29.3 ± 3.30	2.4 ± 0.01	Smooth	Blunt bending	Flat
ST 1	♂	90.6 ± 7.20 a	3.4 ± 0.13 a	Ring thread	Blunt stylus	Dent
ST 2	♂	25.4 ± 3.15 b	2.3 ± 0.08 b	Ring thread	Blunt bending	Dent
SSq	♂	55.0 ± 3.06	3.8 ± 0.34	Vertical grooves	Sharp	Flat
SCh 1	♂	53.5 ± 9.83 a	9.2 ± 0.50 a	Vertical stripes	Sharp	Flat
SCh 2	♂	52.2 ± 2.16 a	4.7 ± 0.02 b	Smooth	Sharp	Flat
SCh 3	♀	21.9 ± 4.69	2.2 ± 0.03	Smooth	Sharp	Flat
SB 1	♂	8.5 ± 0.84 b	1.6 ± 0.03 b	Porous	Blunt	Dent
SB 2	♂	10.2 ± 1.18 a	2.4 ± 0.05 a	Porous	Blunt	Dent
SB 3	♀	13.8 ± 2.07	3.8 ± 0.05	Smooth	Blunt, pore	Extrude
SCo	♂	3.3 ± 0.53	1.5 ± 0.03	Smooth	Finger-like	Dent
SSt	♂	4.5 ± 0.21	2.5 ± 0.02	Smooth	Blunt	Dent
UP	♂	1.4 ± 0.07	1.1 ± 0.03	Porous	Blunt, pore	Dent
SAu 1	♂	11.9 ± 1.23 b	2.0 ± 0.04 a	Porous	Blunt	Dent
SAu 2	♂	13.1 ± 1.65 a	0.9 ± 0.01 c	Porous	Blunt	Dent
SAu 3	♂	8.2 ± 1.05 c	1.2 ± 0.03 b	Porous	Blunt	Dent

Data are presented as mean ± SE.

Different letters indicate *P *< 0.05 in the independent sample *t*-test.

### General Antennal Morphology

The antennae of both male and female *G. menyuanensis* adults were comprised of 3 regions: scape, pedicel, and flagellum (Fig. 6A–H). The scape was relatively thick and short, columnar in shape, with average lengths of 152.7 ± 5.25 μm for males and 33.4 ± 2.56 μm for females (*t *= 3.45; df = 18; *P *< 0.05) (Table 4). The pedicel is cylindrical and shorter than the scape, which connects the distal scape to the basal flagellum (Fig. 6). The average length of the pedicel segment is significantly greater in males (131.8 μm ± 3.60 μm) than in females (109.1 μm ± 8.14 μm) (*t *= 4.38; df = 18; *P *< 0.05). The flagellum comprises 36 to 40 flagellomeres for males (Fig. 6A–C). Each flagellomere has a pair of opposite feathery lateral branches, each ending in 1 to 3 sharp spines (Fig. 6C). The lateral branch and the longitudinal axis of the flagellum of males are at an angle ranging from 30° to 90°. However, the flagellum of adult female *G. menyuanensis* is basiconic-like, curved, and tapering at the top, lacking flagellomere and lateral branches (Fig. 6E–G). The average length of the flagellum is 5957.1 ± 180.66 μm in males and 888.9 ± 15.53 μm in females (*t *= 6.38; df = 18; *P *< 0.05).

The seventh-instar larvae bear a pair of cylindrical, 3-segmented antennae: each consists of a scape, a pedicel, and a flagellum (Fig. 6I–L). The second segment is the longest, while the flagellum is very short.

**Fig. 7.**
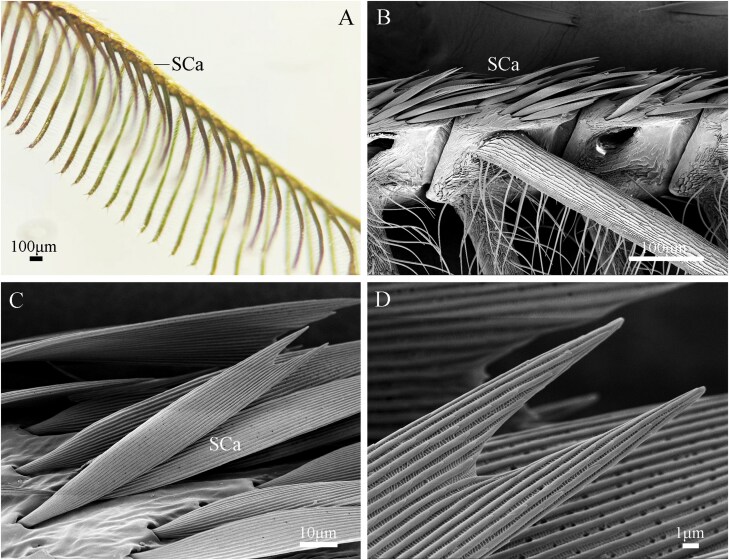
Cuticular modification on the male antennae. (A) Overall view of golden scale (SCa); (B) Lateral view of the flagellomeres; (C) Close-up of the scale on dorsal surface of flagellomeres; (D) A magnified view of scale.

The antennal segments of males are densely covered with golden lamellar scales (Fig. 7A), which are distributed along the dorsal side, covering approximately 60% of the antennal surface area (Fig. 7B). The scales have basal parts inserted into open sockets, with parallel vertical ridges on their surfaces and v-shaped notches at their tips (Fig. 7C, D).

### Types and Distribution of Antennal Sensilla in Male Adults

**Fig. 8.**
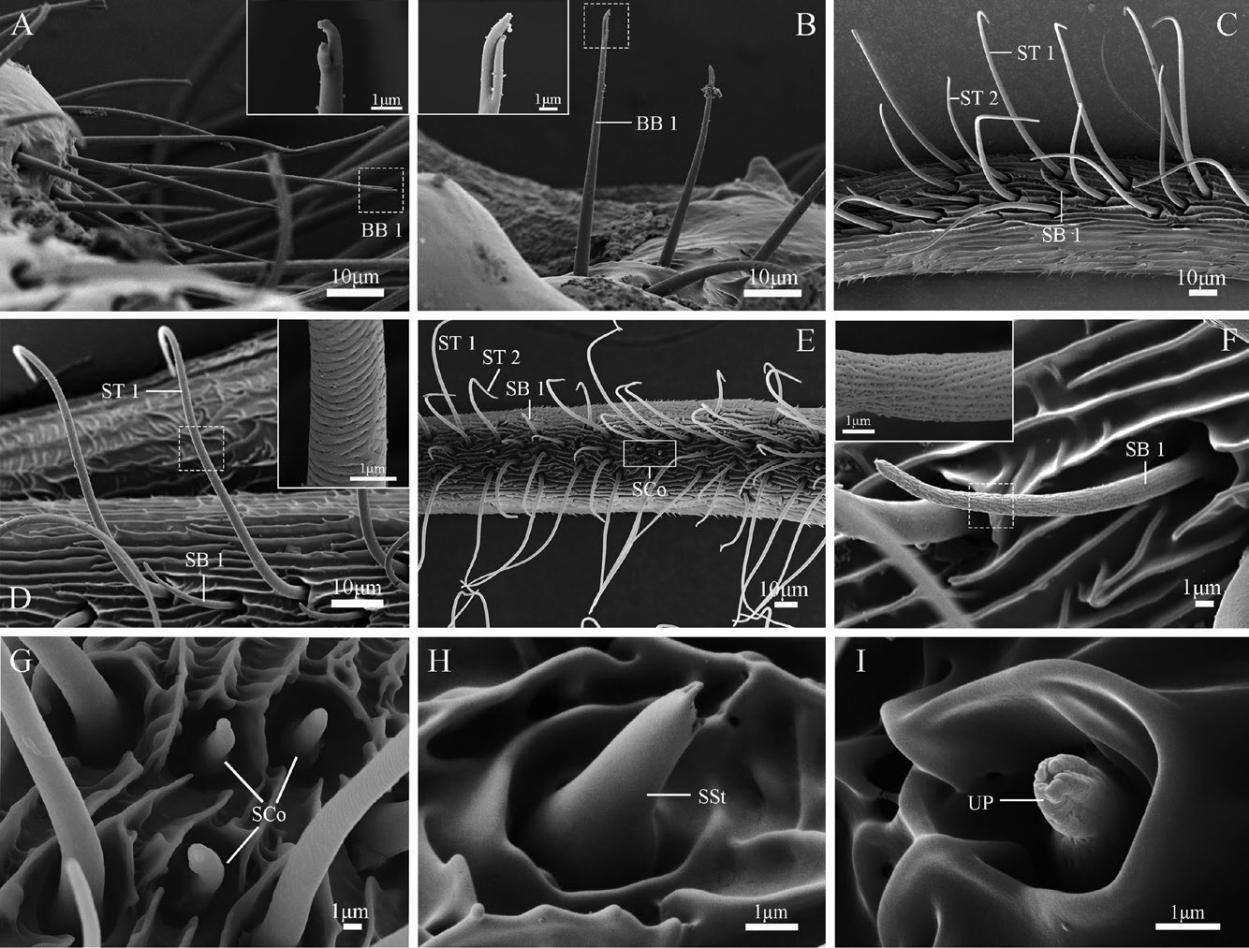
SEM structure of sensilla on the adult male antennae of *Gynaephora menyuanensis*. (A, B) BB 1, Böhm bristles 1; (C) ST 1, ST 2, Sensilla trichodea subtype 1 and 2; (D) Details of the sensilla trichodea (ST 1); (E) Lateral view of male flagellomere showing sensilla trichodea (ST 1, ST 2), sensilla basiconca subtype 1 (SB 1) and sensilla coeloconica (SCo); (F) Details of the sensilla basiconca (SB 1); (G) Details of the sensilla coeloconica (SCo); (H) Details of the sensilla styloconica (SSt); (I) Details of the sensilla uniporous peg (UP).

**Fig. 9.**
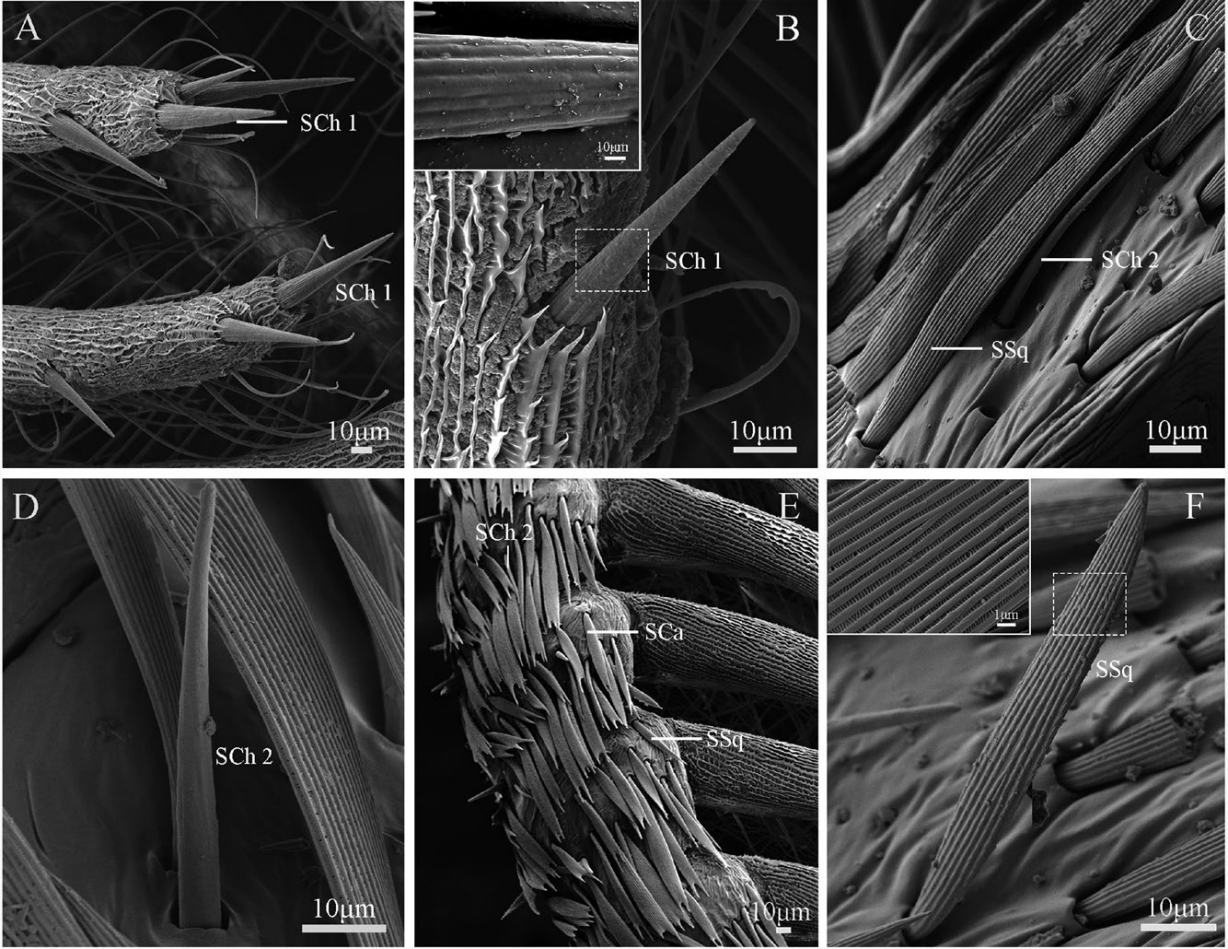
SEM structure of sensilla on the adult male antennae of *Gynaephora menyuanensis*. (A) SCh 1, sensilla chaetica subtype 1; (B) Details of the SCh 1; (C) SSq, sensila squamiformia; SCh 2, sensilla chaetica subtype 2; (D) Details of the SCh 2; (E) SCa, Scale; SSq, senailla squamiformia; (F) Details of the SSq.

**Fig. 10.**
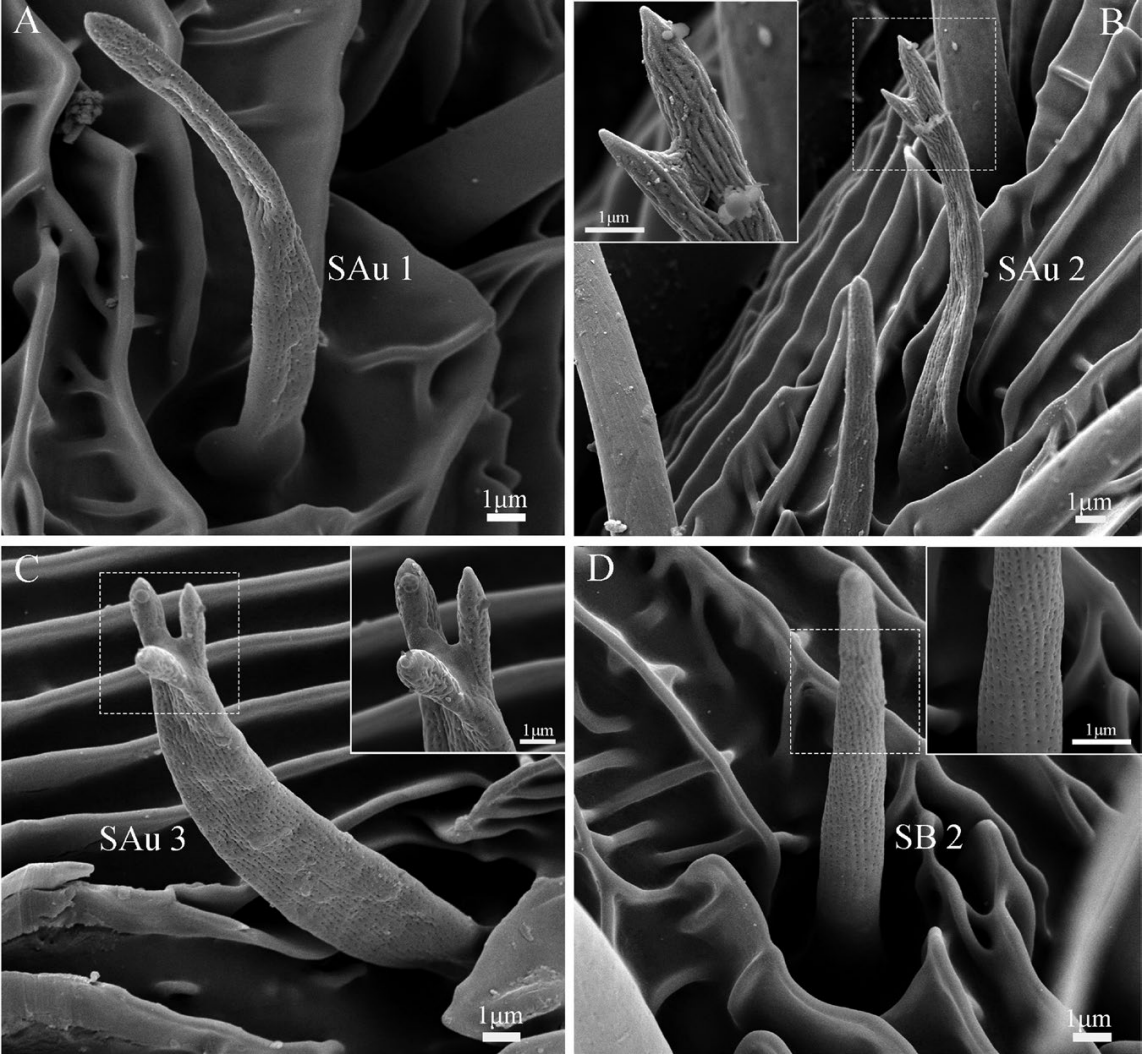
Morphological characteristics of two sensilla on the lateral branches of the bipectinate antennae. (A) Details of the sensilla auicillica subtype 1 (SAu 1); (B) Details of the sensilla auicillica subtype 2 (SAu 2); (C) Details of the sensilla auicillica subtype 3 (SAu 3); (D) Details of the sensilla basiconca subtype 2 (SB 2).

The sensilla are primarily located on the ventral side of the lateral branches of the flagellomeres, with a few on the dorsal side (Figs. 8–10). Nine types of sensilla are identified on male *G. menyuanensis* in this study, namely: Böhm bristles, Sensilla trichodea (ST 1 and ST 2), Sensilla squamiformia, Sensilla chaetica (SCh 1 and SCh 2), Sensilla basiconca (SB 1 and SB 2), Sensilla coeloconica, Sensilla uniporous peg, Sensilla styloconica, Sensilla auricillica (SAu 1, SAu 2 and SAu 3).

#### Böhm Bristles (BB)

Böhm bristles are exclusively observed as agglomerated units on the basal region of the antennae (scape and pedicel) (Fig. 8A, B) and almost perpendicular to the cuticle surface. Böhm bristles are anchored to the basal socket, with an average basal diameter of 3.2 ± 0.18 µm and an average length of 116.4 ± 15.68 µm (Table 5). They gradually taper from the base to the tip, ending in blunt and branched points, and have a smooth, non-porous surface.

#### Sensilla Trichodea (ST)

Sensilla trichodea are observed in the ventral branches of the bipectinate antennae and are the most abundant among the 9 types of sensilla (Fig. 8C). The surface of the sensilla wall has spiral lines and shallow pores; the whole body is slender, and the base has a fossa, which gradually tapers from the base to the tip. Based on their length, sensilla trichodea can be divided into 2 sub types: ST 1, ST 2 (Fig. 8C–E).

Sensilla trichodea 1 are distributed in the center of the ventral lateral branches of bipectinate antennae, with an average length of 90.6 ± 7.20 μm and an average width of 3.4 ± 0.13 μm at the base (Table 5). These sensilla are characterized by their short and hair-like structure with a slight curvature at the blunt and rounded top (Fig. 8C, D). Sensilla trichodea 2 are found mainly on the ventral and distal branches of the antennomeres and exhibits a pronounced degree of curvature toward the tip, with a larger bending angle compared to sensilla trichodea 1 (Fig. 8D). The average length of sensilla trichodea 2 is 25.4 ± 3.15 μm, with a significant difference between the 2 subtypes (*n* = 25; *t* = 0.84; df = 48; *P* < 0.05).

#### Sensilla Basiconca (SB)

Sensilla basiconca are mainly distributed in the depression on the ventral surfaces of the flagellomeres (Figs. 8C–F and 10D). Based on its shape and morphological features, sensilla basiconca can be divided into 2 subtypes. Subtype 1 (SB 1) is characterized by a short, hair-like structure with a slight curvature at the blunt and rounded top (Fig. 8C–F). The surface is covered with small holes. SB 1 is usually positioned at an angle between 30° and 45° to the lateral branches. In males, SB 1 have an average length of 8.5 ± 0.84 µm and a base width of 1.6 ± 0.03 µm (Table 5).

Sensilla basiconca 2 (SB 2) is located on the lateral branches of the bipectinate antennae and are obviously longer than SB 1 sensilla (*t *= 0.23; df = 48; *P *< 0.05) (Fig. 10D). These sensilla are characterized by a conical shape with a wide base and a blunt tip. The cuticular surface bears longitudinal ridges with numerous pores between the ridges. SB 2 has an average length of 10.2 ± 1.18 µm and a base width of 2.4 ± 0.05 µm (Table 5).

#### Sensilla Coeloconica (SCo)

Sensilla coeloconica are located in a rounded, shallow cavity formed by the concave antennal epidermis and are flower-shaped (Fig. 8E, G). They occur either individually or in clusters of 2–4. The cavity features a central cone that stands upright in the middle, with a smooth surface and blunt tip. The average length is 3.3 ± 0.53 µm and the average width of the base is 1.5 ± 0.03 µm (Table 5).

#### Sensilla Styloconica (SSt)

Only a single sensilla styloconica is located at the apex of the lateral branches of each flagellomere and slopes toward the antenna’s surface with an angle of 30º to 45º. These sensilla are characterized by a conical shape with a wide base and rabbit-ears tips. The cuticular surface is smooth (Fig. 8H). The length of the sensillum is measured at 4.5 ± 0.21 µm and the average basal diameter is 2.5 ± 0.02 µm (Table 5).

#### Sensilla Uniporous Peg (UP)

The sensillum uniporosum peg is an epidermal specialization and is primarily found on the lateral and ventral sides of the flagellomeres (Fig. 8I). Only 2 to 3 UPs are present in the antennae of male adults. The papillate UPs are embedded in deep sockets and have an enlarged base with a small hole at the end, along with longitudinal notches surrounding the hole. On average, the UP sensillum is 1.4 ± 0.07 µm long and 1.1 ± 0.03 µm wide (Table 5).

#### Sensilla Chaetica (SCh)

Two subtypes of sensilla chaetica are identified, namely sensilla chaetica1 (SCh 1) and sensilla chaetica 2 (SCh 2) (Fig. 9A–D). SCh 1 are distributed at the apex of the lateral branches of each flagellomere, with 3 or 4 sensilla per subsegment (Fig. 9A). The SCh 1 resemble an upright spine with a thicker base and no socket-like depression. Their outer walls exhibit longitudinal lines and sporadic pores, and the tips are blunt and rounded (Fig. 9B). The average length of the sensilla is approximately 53.5 ± 9.83 µm, and the average width at the base is 9.2 ± 0.50 µm (Table 5).

Sensilla chaetica 2 are present on the dorsal side of the flagellum, adjacent to sensilla squamiformia, and covered by scales (Fig. 9C). These sensilla have a sickle-shaped structure with a smooth surface, curving toward the tip (Fig. 9D). The average basal diameter of these sensilla is 4.7 ± 0.02 µm and the average length is 52.2 ± 2.16 µm, which is longer than SCh 1 (*t *= 4.27; df = 48; *P *= 0.015) (Table 5).

#### Sensilla Squamiformia (SSq)

Sensilla squamiformia resemble slender scales. They are scabbard-shaped, with longitudinal cuticular grooves on the surface, and slightly curved from the middle region with sharp distal tips and no obvious depressions (Fig. 9E, F). They are randomly interspersed among the scales on the scape, pedicel, and flagellomeres. The average length of the scaly sensilla in males is 55.0 ± 3.06 μm, and the average width of the base is 3.8 ± 0.34 μm (Table 5).

#### Sensilla Auricillica (SAu)

Sensilla auricillicaare located on the ventral and dorsal surfaces of the feathered branch of the antennae (Fig. 10A–C). They arise from socket-like depressions, with a grooved and porous surface. Three subtypes of SAus, namely SAu 1, SAu 2, and SAu 3 were identified based on their morphology.

Sensilla auricillica subtypes 1 (SAu 1), with an average length of 11.9 ± 1.23 µm and an average base width of 2.0 ± 0.04 µm, exhibit a leaf-like shape, resembling that of gramineous plants, curved at the middle regions and tapering apically to a blunt end (Fig. 10A). Subtype 2 are notable longer than SAu 1 and SAu 3 (*F =* 3.94; df = 2, 72; *P *< 0.05), with an average length of 13.1 ± 1.65 µm and an average base width of 0.9 ± 0.01 µm (Table 5). The overall shape of SAu 2 resembles that of rabbit ears with 2 fork-shaped branches at the distal end (Fig. 10B). The SAu 3 sensilla are slightly shorter than SAu 1 and have a glove-like structure with a narrowed base that gradually widens towards the top, where there are 1 or 3 pointed protrusions (Fig. 10C). The average length is 8.2 ± 1.05 µm, and the average width of the base is 1.2 ± 0.03 µm (Table 5).

### Types and Distribution of Antennal Sensilla in Female Adults

**Fig. 11.**
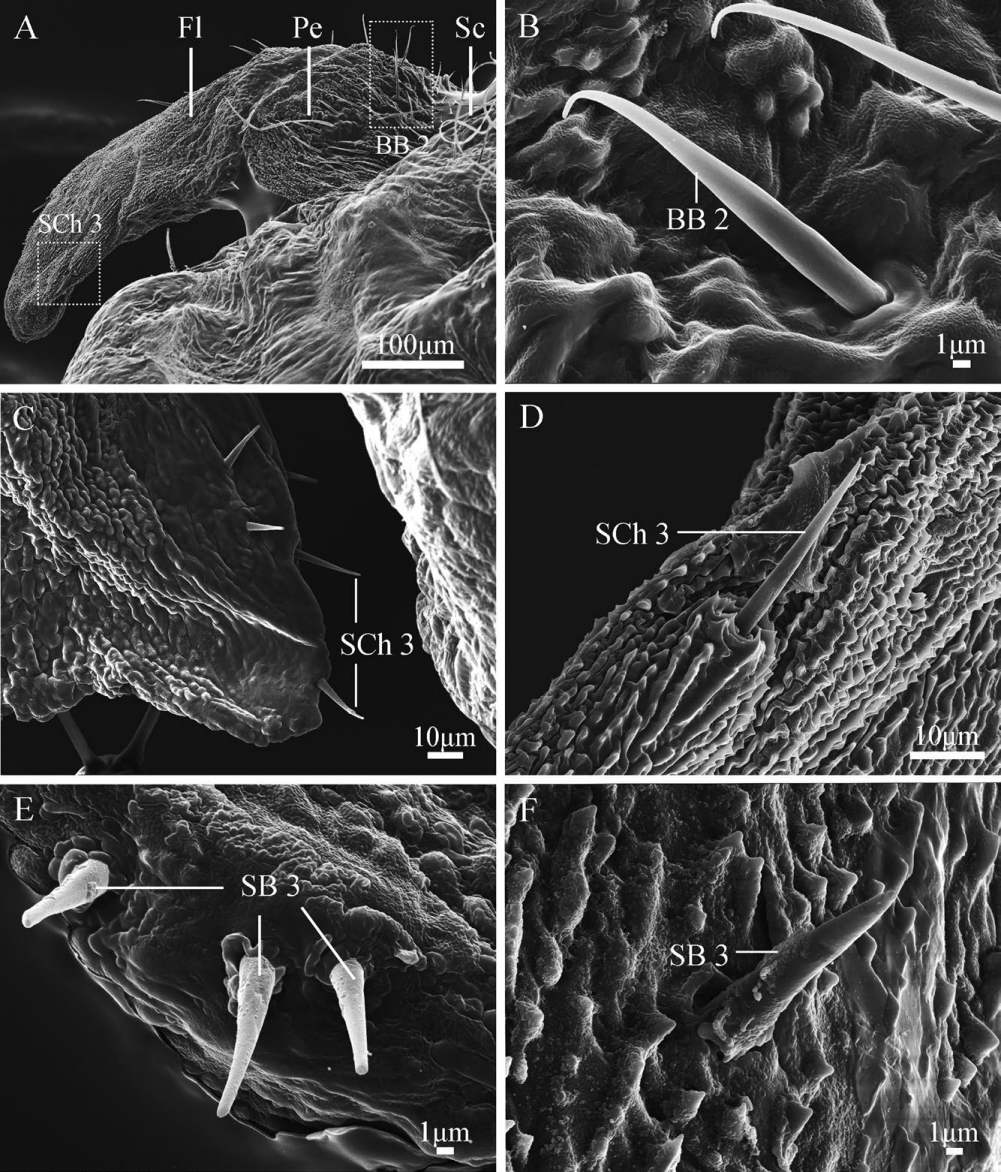
SEM structure of sensilla on the adult female antennae of *Gynaephora menyuanensis*. (A) scape (Sc), pedicel (Pe), and flagellum (Fl) of female; (B) BB 2, Bǒhm bristles on the antenna of the female; (C, D) SCh 3, Sensilla chaetica subtype 3; (E, F) SB 3, Sensilla basiconica subtype 3.

Three types of antennal sensilla, including Böhm bristles 2 (BB 2), Sensilla chaetica 3 (SCh 3), and Sensilla basiconca 3 (SB 3) are observed on female *G. menyuanensis* (Fig. 11A). Böhm bristles 2 (BB 2) are located as agglomerated units at the bases of the scape and pedicel, and exhibit a long, hair-like shape, tapering from the lower to the upper end. The tips of these sensilla are sharp with a pronounced degree of curvature, and the surface is smooth without longitudinal lines (Fig. 11B). The base of BB 2 inserts into a round depression and the average width is 2.4 ± 0.01 µm (Table 5). Sensilla chaetica (SCh 3) exhibit a straight shape with a smooth surface and a blunt tip (Fig. 11C, D). It is sparsely positioned at the apex of the flagellomeres. The average length is 21.9 ± 4.69 µm and the average width of the base is 2.2 ± 0.03 µm (Table 5). Sensilla basiconca (SB 3) are located on the side of the flagellomeres and is characterized by its upright structure, flat distal ends, and smooth surface with abundant thorns (Fig. 11E, F). The average length is 13.8 ± 2.07 µm and the average width of the base is 3.8 ± 0.05 µm (Table 5).

## Discussion

Although 8 species of *Gynaephora* have been identified in China, this is the first time the life history, and the morphology of the eggs, mature larvae, pupae, and antennae of male and female adults have been reported. *G. menyuanensis* completes its life cycle on the Qinghai-Tibet Plateau (QTP), contributing to the deterioration of alpine meadows. Knowledge of the development time is important for designing control measures because this could target the early stages of development. The time needed for the full development from egg to adult stage of *G. menyuanensis* was about 350 d, significantly longer than other Lepidopterous insets, such as *Plutella xylostella*, *Spodoptera frugiperda,* etc. (Kulkarni and Evenden 2024, Lu et al. 2024). This pest can remain under the roots or in the soil for up to 230 d during the emergence period, which should be considered in management strategies.

Determining larval stages in grassland caterpillars plays a key role in describing the age distribution of populations, predicting potential dispersal, and integrated pest management. Traditional studies on instar determination have been based on direct observations of specific external morphological indices of the insect body and determined the larval instar using the frequency distribution method. This approach can yield some bias if the overlap area of 2 normal distributions is too large (McClellan and Logan 1994, Esperk et al. 2007a, 2007b). Determining the number of instars using cluster analyses successfully addresses these problems (Wu et al. 2013, Yang et al. 2018, Wang et al. 2022). Our study constitutes the first multivariate approach to identify the larval instar number in grassland caterpillars. Through multivariate analysis of 4 morphometric traits, 7 groups were identified which are equivalent to 7 larval instars. Our study provides an excellent example of the use of *k*-means clustering in entomological taxonomy. Validation showed that the growth trajectory of the 7 larval instars of *G. menyuanensis* aligned more closely with the Brooks-Dyar rule. The *Cn* index for larva head width was less than 0.1 (Table 1), and the *Cn* index for body length, body width, and number of crochets exceeded 0.1, indicating the rationality of the 7-instar hypothesis. An unusual phenomenon is that the female larvae might have a final stage lasting 10 to 15 d longer than male larvae (Table 3). We speculate that the female larvae might have one more instar than the male larvae. Therefore, further studies should focus on verifying this hypothesis.

Typically, the types, distribution, quantities, and functions of antennal sensilla are relatively stable among the same taxonomic groups. Especially in Lepidoptera, male and female antennae generally have 8 sensilla types. However, in our study, significant differences were found in the general antennal length (the male antennae were obviously longer than those of the females) and in the types, numbers, and distribution of sensilla between males and females (Tables 4 and 5; Fig. 6). Male adult antennae have 9 types and 14 subtypes of sensilla, while 3 types of sensilla are observed in the female adult antennae. Previous studies have reported that the antennae of male adults have more diverse types of sensilla and are longer than those of females across different insect species, such as *Erannis ankeraria* Staudinger (Liu et al. 2019) and *Popillia japonica* Newmann (Kim and Leal 2000). These findings underscore the significance of male antenna as crucial sensory organs because they need more sensilla to detect sex pheromones and locate females. Based on our observation, the female *G. menyuanensis* lack wings and legs, so the antennal olfactory system of males must house abundant and specialized neurons that respond electrophysiologically to sex pheromones when searching for mates (Tim et al. 2021).

The types antennal sensilla in *G. menyuanensis* observed in this study encompassed chemical sensilla, mechanical sensilla, taste sensilla, temperature and humidity sensilla, and more. There are 4 types of antennal chemosensory sensilla, including sensilla coeloconica, sensilla basiconica, sensilla trichoidea, and sensilla auricillia. Among these, sensilla trichoidea and sensilla auricillia are the most complex sensilla observed on male *G. menyuanensis* antennae. Sensilla trichoidea showed the most prevalent distribution in our study and are morphologically similar to other lepidopteran insects (Gómez et al. 2003, Chang et al. 2015, Wang et al. 2022). Presumably, the absence of a socket would restrict mechanosensation, while the long hair-shaped and numerous pores on the walls are related to perceive sex pheromones and plant odors. Observations on *Diaphania angustalis* and *Erannis ankeraria* Staudinger, 1861 showed that sensilla trichoidea were abounding in male antennae yet sparse on female antennae, demonstrating their olfactory functions (Liu et al. 2019, Zhang et al. 2019). Molecular studies on other species have validated the abundance of pheromone-binding protein and olfactory receptors in sensilla trichoidea, indicating role in detecting pheromones and plant volatiles (Zhang et al. 2017b, Cloonan et al. 2020).

Sensilla auricillica in *G. menyuanensis* has a rabbit-ear or tongue profile and a multiporous surface, similar to many other Lepidoptera. For example, *Diaphania angustalis* Snellen (Zhang et al. 2019) and *Spodoptera frugiperda* (Wang et al. 2023b) have one subtype of SAu. *Paranthrene tabaniformis* has 2 subtypes of SAu (Zheng et al. 2023). However, we noted 3 subtypes of sensilla auricillica in *G. menyuanensis*. This porous structure on the antennae may be related to the detection of sex pheromones and host volatiles (Ansebo et al. 2005, Faucheux et al. 2006). Both the female and male antennae of *G. menyuanensis* were found to have sensilla basiconca, consistent with other lepidopteran insects (Seada 2015, Bawin et al. 2017, Li et al. 2024). However, the total number of sensilla was obviously greater in males than females in our study, similar to those reported in *Cydia pomonella* and *C. succedana* (Roh et al. 2016) and *Earias vittella* (Rani et al. 2021). The external morphology of SB on the male antennae was also similar to that observed in studies carried out on *Earias vittella* (Rani et al. 2021), *Helicoverpa armigera* (Abalavadi Thammaiah et al. 2022) and *Spodoptera frugiperda* (Li et al. 2024). Typically, sensilla basiconica are perforated by numerous small pores indicating their olfactory chemoreceptive function (Li et al. 2024). However, in our study, we found that sensilla basiconca 3 on the female adult antennae had a smooth wall with abundant thorns (Fig. 11E, F). Since the common role of sensilla basiconca is associated with detecting host plant cues, mostly relevant for females, we speculate the sensilla basiconca 3 might play a similar role and further research is needed to verify.

Three types of mechanical sensilla are observed, including Böhm bristles, sensilla chaetica and sensilla squamiformia, which can detect external mechanical stimuli. Böhm bristles are spine-like, aporous sensilla exclusively found on the scape and pedicel of filiform antennae, suggesting that an association with the mechanical rotation of the antennae (Zacharuk 1980, Krishnan et al. 2012). The straight, rod-like structural features of sensilla chaetica are corroborated in our investigation on the ultrastructure of the antennae of *G. menyuanensis*. Previous studies reported that SCh might play a dual role in both mechanical and chemical perception because they not only respond to mechanical shocks and play a crucial role in selecting suitable sites, behavioral environments, and courtship microenvironments, but also have a terminal sensory pore at the tip that enables chemical perception (Dong et al. 2020). However, it is not known whether sensilla chaetica in *G. menyuanensis* possesses a chemosensory function due to the absence of a pole in our investigated samples.

The temperature and humidity sensing abilities of insects are related to sensilla styloconica, sensilla coeloconica, and uniporous peg sensilla (Ren et al. 2014). Sensilla styloconica, typically non-porous, can sense temperature and humidity and is widely distributed on the antennae of Lepidoptera, such as *Copitarsia consueta* Walker and *Plutella xylostella* (Castrejón-Gómez et al. 1999, Wee et al. 2016). Sensilla coeloconica is only present on the ventral surface of the flagellum in male *G. menyuanensis* and may be involved in perceiving the temperature and humidity changes in the habitat (Ruchty et al. 2009). In the male antenna of *G. menyuanensis*, the uniporous peg sensilla were identified for the first time. This sensilla type was described in the subfamilies of Noctuidae and Pyralidae (Faucheux 1990, Guo et al. 2022). This is the first time they have been observed in the family Lymantridae. Previous researchers indicated that sensilla uniporous peg had no olfactory functions and suggested that these sensilla potentially sense humidity or carbon dioxide sensitivity (Rani et al. 2021, Wang et al. 2023b). Therefore, future investigations should focus on elucidating the specific functions of these sensilla types and their underlying molecular mechanisms. As the grassland ecosystems are severely degraded by *G. menyuanensis*, determining larval instars and studying their life history traits is vital for prompt and effective control applications. The number, size, and arrangement of antennal sensilla differ from those of caterpillars and can be used to identify this endemic species. Our findings provide a scientific foundation for exploring new strategies in pest control.

## Supplementary Material

ieaf006_suppl_Supplementary_Figures_S1

ieaf006_suppl_Supplementary_Figures_S2

ieaf006_suppl_Supplementary_Figures_S3
