# Supplementary figures and images for: Instar determination, development, and sexual dimorphism for Gynaephora menyuanensis (Lepidoptera: Lymantriinae) and ultrastructure of adult antennae
Source: J Insect Sci. 2025 Mar 14;25(2):1. doi: 10.1093/jisesa/ieaf006 (PMC11908424; doi:10.1093/jisesa/ieaf006)

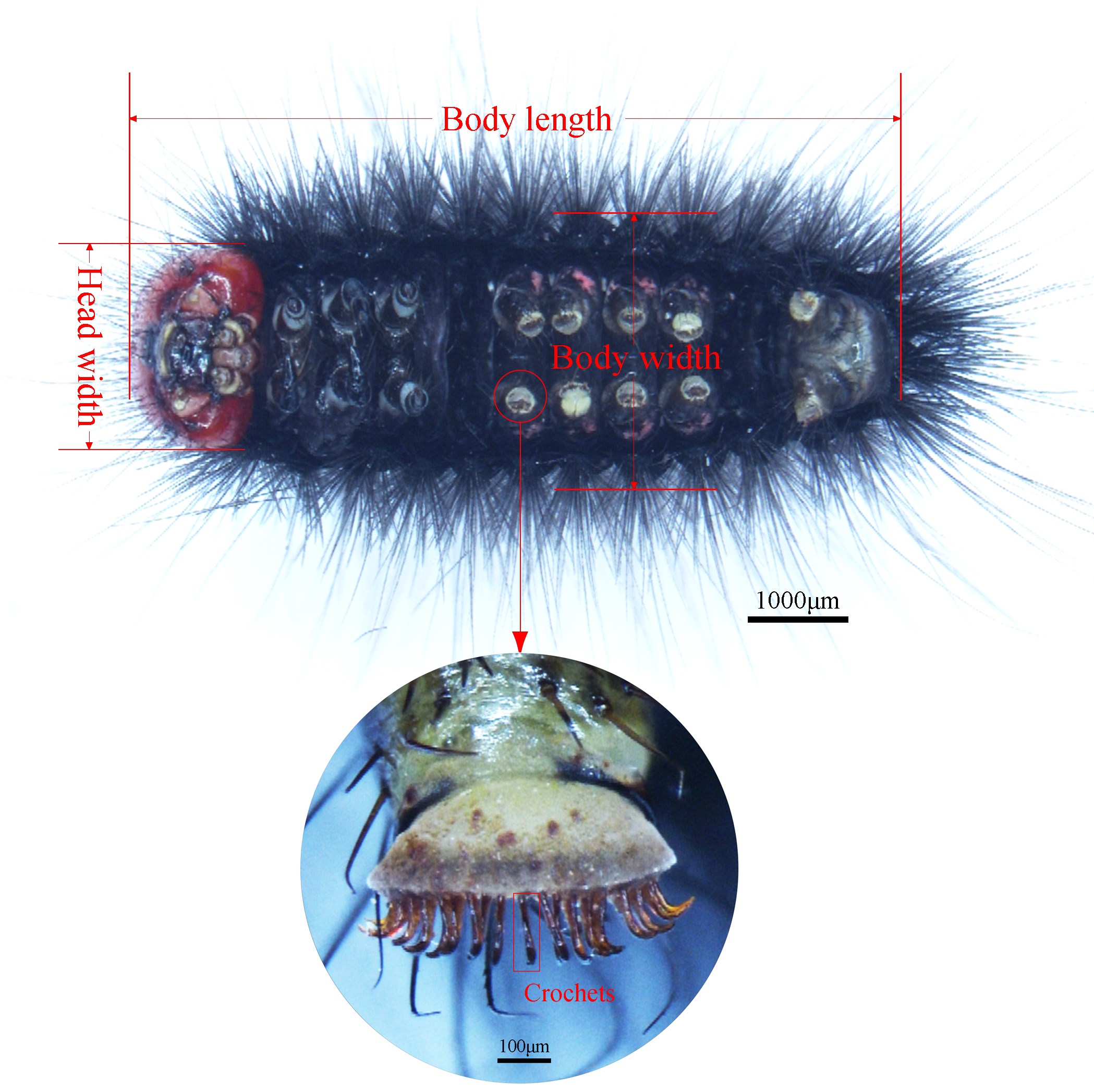

Supplement: ieaf006_suppl_Supplementary_Figures_S1 [file ieaf006_suppl_supplementary_figures_s1.jpeg]

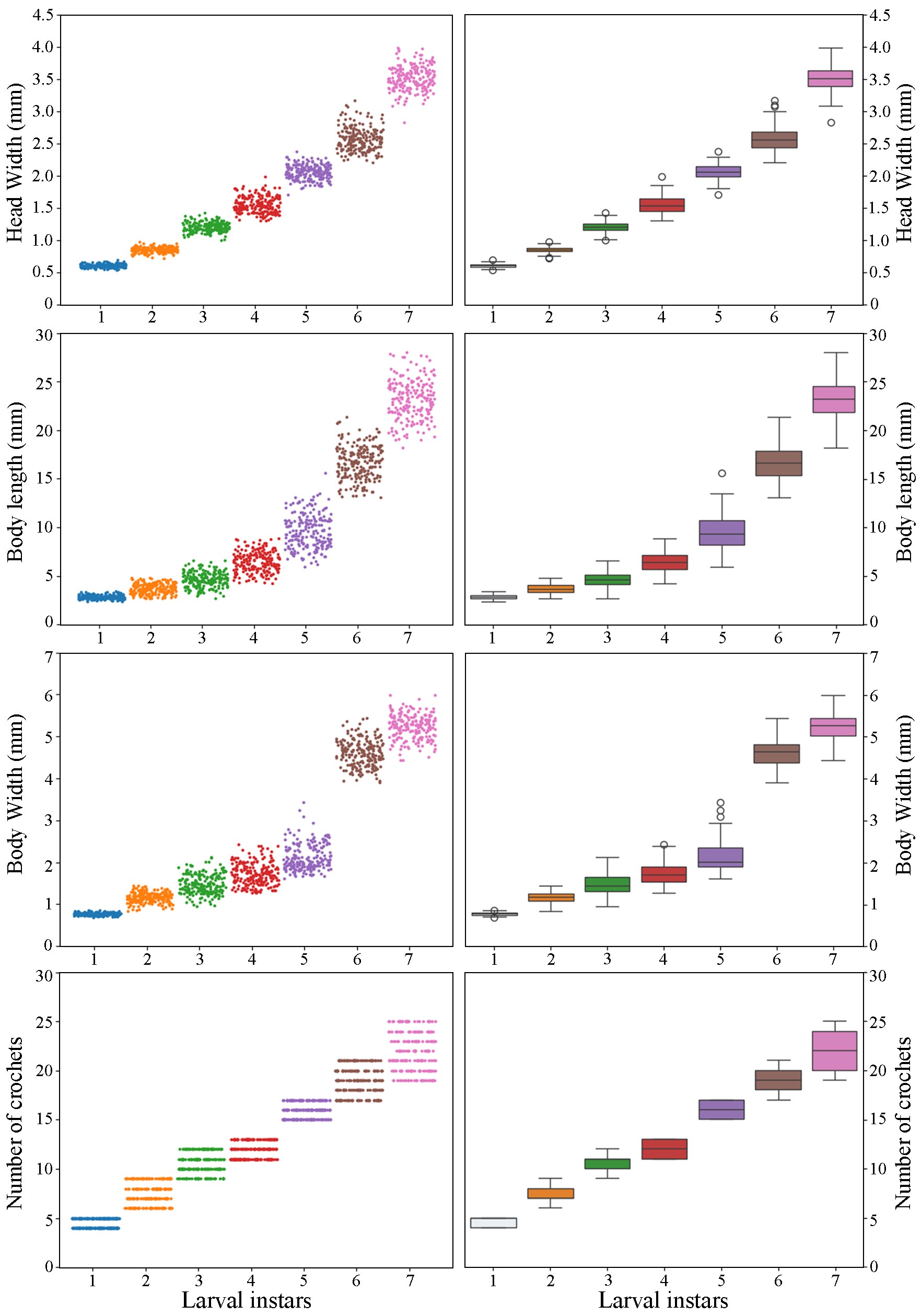

Supplement: ieaf006_suppl_Supplementary_Figures_S2 [file ieaf006_suppl_supplementary_figures_s2.jpeg]

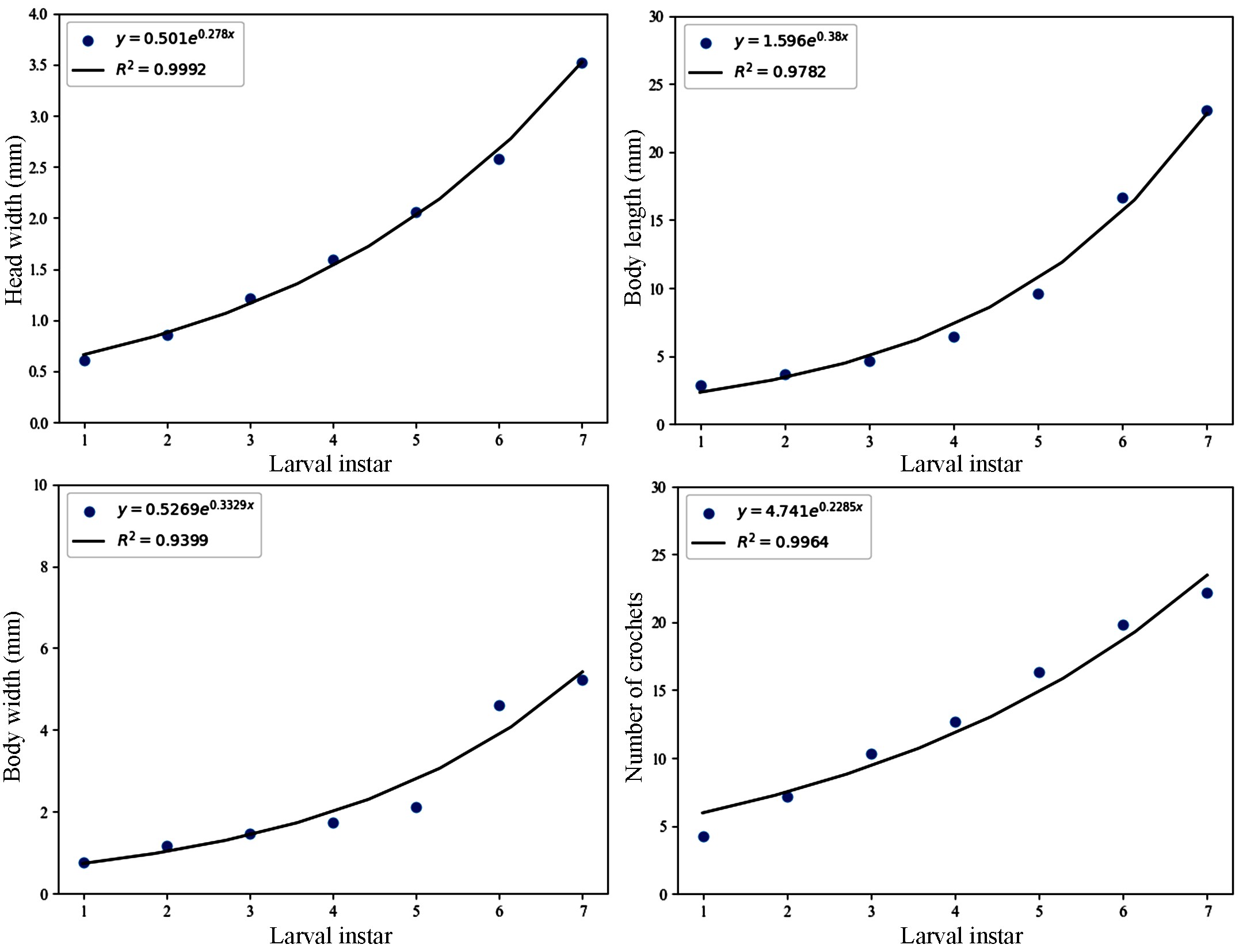

Supplement: ieaf006_suppl_Supplementary_Figures_S3 [file ieaf006_suppl_supplementary_figures_s3.jpeg]
